# A modified systematic review of research evidence about education for pre-registration nurses in palliative care

**DOI:** 10.1186/1472-684X-13-56

**Published:** 2014-12-10

**Authors:** Nahyeni Bassah, Jane Seymour, Karen Cox

**Affiliations:** School of Health Sciences, Queen’s Medical Centre, University of Nottingham, Nottingham, UK

**Keywords:** Death and dying, Palliative care, Nursing education, Preregistration nursing, Resource-poor countries, End of life care

## Abstract

**Background:**

We undertook a modified systematic review of research regarding educational approaches to and effectiveness of pre-registration palliative care nursing, to inform the development of a short course in palliative care for pre-registration nursing students in Cameroon. The aim of this review was to examine educational approaches applied to pre-registration palliative care nursing education and their effectiveness, and to discuss implications for the development of palliative care curricula in resource-poor countries.

**Method:**

A modified systematic review of research on palliative care educational interventions, conducted with pre-registration student nurses was undertaken. Relevant literature was gathered from CINAHL, EMBASE, MEDLINE and PsychINFO databases for the period 2000–2013. Inclusion was limited to studies of educational interventions evaluating the effectiveness and outcomes of palliative and end of life care education with pre-registration student nurses.

**Results:**

17 studies were found, all of which were conducted in resource-rich countries: United States of America, Canada, Australia, and United Kingdom. Palliative care nursing education at pre-registration level is either delivered as a discrete course within the curriculum or palliative care content is embedded into other nursing specialty courses throughout the wider curriculum. Palliative care education is delivered to students at a variety of stages in their nursing program, using a mix of both didactic and experiential educational strategies. Course facilitators span palliative care specialists, educators who have attended ‘train-the-trainer’ courses in palliative care, and nurses with hospice experience. Education is underpinned by transformative and experiential learning theories and reported as effective in improving students’ attitudes towards care of the dying.

**Conclusion:**

The educational strategies identified in this review may be applicable to resource-poor countries. However, there are challenges in transferability because of the lack of availability of specialist palliative care practitioners who can serve as educators, specialist palliative care units/institutions for experiential learning, funds to design and use high fidelity simulations, and palliative care textbooks and other educational materials. There is thus a need for innovative educational strategies that can bridge these barriers in resource-poor countries. There is also a need for further research into how palliative care education impacts on pre-registration student nurses’ knowledge and practice.

## Background

Nurses play a fundamental role in the care of patients with life-threatening illnesses and end of life care is a part of every nurse’s’ every day practice [1, 2]. This means it is essential that all nurses are competent and feel confident in applying palliative care in their clinical practice. However, existing research identifies a lack of palliative care knowledge and skills among practising nurses, both newly graduated and student nurses [3–5]. While qualified nurses report that they have not received adequate palliative care education [6, 7], newly graduated nurses report a lack of competence and confidence to care for patients and their families in palliative care situations [4, 8]. Moreover, some preregistration nursing curricula do not include any palliative care content [9–11] and preregistration students have been found to have negative attitudes towards death and the care of the dying, expressing feelings of hesitancy, and anxiousness, and being unprepared and untrained to care for a dying person [12–14].

Although palliative care is beginning to feature in pre-registration nursing curricula in some resource-rich countries [4, 10], there is still a significant lack in the curricula of resource-poor countries [3, 9, 11]. By ‘resource-poor’ countries, we agree with the classification made by Wright (2003) (cited in Hunt, 2008:680) [15], who combines the following indices of gross domestic product: health expenditure, overall health system achievement, human development and morphine consumption, to identify resource-poor countries in relation to health. Therefore Sub-Saharan African countries, India and Russia that have a limited potential to provide palliative care are considered to be resource-poor.

This paper presents a modified systematic review which aimed to:

Report on approaches that have been employed to educate pre-registration nurses about palliative care.

Examine evidence of the effectiveness of this education.

Discuss implications for the development of palliative care curricula in resource-poor countries.

## Method

While a traditional systematic review demands a predetermined protocol and entails the identification, evaluation and interpretation of available research regarding a precise question, using explicit search methods, inclusion criteria, as well as a data extraction strategy and scientific quality appraisal tool [16], a modified review might not include all of these [17]. In this modified review, we predetermined the search terms and the research aims and inclusion criteria were clearly stated. We included both studies that employed quantitative, qualitative or mixed methods. In addition, we did not use any instrument to formally assess the scientific quality of the studies included. This modification was informed by the dearth of research about this topic.

### Searches

We searched the following databases: CINAHL, EMBASE, Medline, and PsychINFO for journal articles published in the English language from January 2000 to December 2013. We employed the following terms in our search: palliative care, end of life care, death and dying, care of the dying, and terminal care, in combination with the Boolean operator (AND) nursing education. In addition, we checked the references listed in identified articles.

### Inclusion criteria for articles

**Figure 1:**
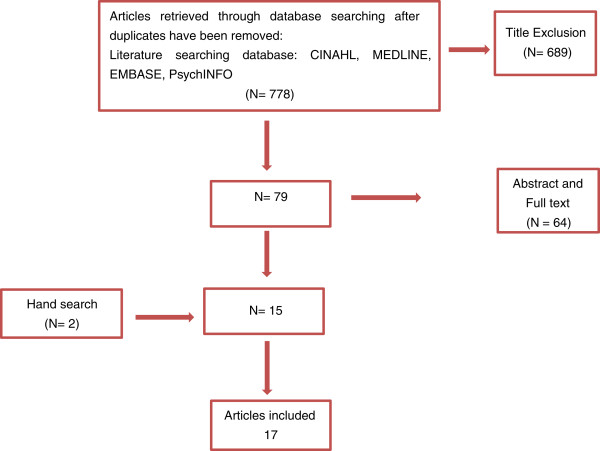
**PRISMA flow diagram.**

After obtaining the articles, we checked their titles and abstracts and later the full texts to see if the inclusion criteria were met (Figure 1). We included quantitative, qualitative and mixed methods articles if:

They reported an educational intervention aimed to improve students’ palliative and end of life care competencies.

They related to the education of pre-registration student nurses or a multidisciplinary group of undergraduate students, including student nurses.

They were published in the English Language.

We excluded educational intervention studies focusing on the education of undergraduate student nurses on paediatric palliative care or end of life communication skills only, as well as descriptive studies of a palliative and end of life care educational approach not aimed at improving student nurses’ palliative and end of life care competencies.

### Data extraction, analysis and synthesis

We extracted data about the following: place and year of study, characteristics of study participants, aims and objectives of the intervention, contents and strategies used in the intervention, evaluation methodology and reported outcomes and effectiveness. Data analysis and synthesis were guided by the review aims and results are presented under main themes as shown in Table 1.

**Table 1:** **Summary table of educational strategies, evaluation methods and effectiveness**

Author, year and country	Course aims/objective	Educational methods	Study design	Study outcomes and effectiveness
**Arber** [18] **UK**	To measure 3^rd^ year students’ knowledge of palliative care before and after a palliative care module	Lecture, and hospice practicum experience	Pretest-posttest	Statistical significant (P = 0.001) increase in knowledge
**Frommelt** [19] **USA**	To examine the effects of education on attitude towards care of the dying patient and their families	Lecture, Role play, case study presentations in story formats,	Controlled Pretest-posttest	Significant positive change in attitude for the intervention group t = 7.283, p <0.05
**Mallory** [20] **USA**	To assess the impact of education on attitudes towards care of the dying	Lectures, group discussions, Role play, visit to a gross anatomy cadaver laboratory, funeral home and hospice	Controlled pretest- posttest longitudinal	Significant improvements (p < .05) in attitude score in the intervention group
**Thompson** [21] **USA**	To assess the degree of change in comfort level in the care of the dying that students experience during a palliative care course	Interactive lectures, online discussion, reading of recommended books, field trips to a funeral home, role playing, journaling of experiences	Pretest-posttest	Increase in comfort levels from an average of 4.8 before to 7. 5 after
**Kwekkeboom et al.** [22] **USA**	To assess the impact of a volunteer companion program on students’ knowledge, attitudes and concern caring for a dying patient	Orientation lecture, spending time with patients and their families, making up bereavement phone calls to families of patients previously cared for, keeping Journal of experiences	Controlled pretest- posttest	No significant change in knowledge; Significant decrease in concerns scores P <0.01 Attitude scale not assessed due to poor reliability
**Brien et al.** [23] **Canada**	To develop effective nursing competencies for end of life care among undergraduate student nurses.	Plenary reflective activities, viewing of a documentary film and participating in role-playing simulations.	Mixed Method	Attitudes related to apprehensions and beliefs regarding interventions with dying persons and their families changed positively for most students.
**Barrere et al.** [24] **USA**	To assess the influence of integrating the ELNEC curriculum into a 1 year and 4 years baccalaureate program on students’ attitudes toward care of the dying	Lecture, discussions, clinical placement experience in the care of the dying in hospitals, homes, extended care facilities and hospices	Pretest-posttest	Type of program not significant in attitude change. Significant improvements in attitudes, t = -5.977, p = .000
**Leighton et al.** [13] **USA**	To facilitate application of theory content to a clinical end-of-life scenario.	Simulated end of life care clinical experience.	Qualitative	Helped students to look at grief in a different way, students found interacting with grieving family members to be rewarding Real nature of the simulation enhanced students’ learning.
**Dibartolo et al.** [25] **USA**	To enhance student knowledge of the dying process and the complexity of human reactions when facing EOL situations	An assignment using Cinemeducation approach in which students were asked to view either of these two films: *Tuesdays with Morrie or Whose Life Is It Anyway?* and to answer some questions relating to the film as well as describe lessons learned from the film that could be used in their practice in similar situations	Not clear	Most students felt assignment was helpful in identifying pertinent issues about end of life care. The responses to the assignment questions submitted by students were good in quality and insight with grades ranging from A to D, and a majority with B. Students could identify the stages of grief.
**Weismann et al.** [26] **USA**	To examine effect of ENLEC communication module on first years ADN students self-efficacy in communication skills	Lecture, group discussion, Role play Listening to a classmate describe a significant loss in his/her life,case study	Controlled pretest-posttest	Significant improvements in the intervention group on both the scores on the VAS and FATCOD. Also there was improvements in the controls group’s score on the VAS and FATCOD, indicating both approaches are effective
	To determine if a specially prepared palliative care module and an embedded content course lead to positive attitude towards care of the dying patient			
**Dobbin** [27] **USA**	To assess the impact of an elective nursing course incorporating the ENLEC curriculum content on associate degree student nurses’ attitudes towards death and care of the dying	Lecture with PowerPoint slides (for students in the intervention groups) supplemental text, visit to a hospice and funeral home Watching of the film ‘Wit’ (students in elective course), reference palliative care content of a medical-surgical book	Controlled pretest-posttest	Both content delivery methods were found to change students’ attitude towards death to an extent. However, students who watched the film Wit recorded significant positive changes
	To evaluate the effect of an end-of-life module embedded in a larger course on the attitudes of a similar group of students.			
**Gilliland** [28] **USA**	To examine the effect of a planned clinical experience with dying patients on student attitudes and self-perceived competencies in end-of-life care.	2 days hospice experience	pretest-posttest	Significant change in attitude. No significant change in self-reported end of life care competencies
**Bush T** [29] **Australia**	To determine if the completion of a palliative care elective aided nursing students in the clinical provision of palliative care	Lectures and end of life care simulation	Posttest-only	Students reported that the course was beneficial to their learning about end of life care and appreciated the inclusion of palliative care in their program
**Eaton et al.** [30] **USA**	To explore the perceived influence of an end of life care simulation on students’ learning in home health and hospice practicum setting	End of life care simulations and debriefing session	Descriptive Phenomenology	Students reported that gaining experience in a safe environment prior to practice placement had a positive impact
**Moreland et al.** [31] **USA**	To evaluate the effect of a 15-minute simulation involving a terminally ill lung cancer patient on student nurses knowledge of end-of-life signs and symptoms and perceived self-efficacy	End of life care simulations and debriefing session	Mixed method	11% increase in overall knowledge and self-efficacy improved significantly post-simulation from 35.36 to 37.79 (p = .05). Students expressed difficulty with changing their perspectives from curing to caring for the dying client.
**Fluharty et al.** [32] **USA**	To assess whether there will be an increase in students’ end of life care knowledge, self-confidence in the care of the dying and self-reported communication skills in working with end of life patients after participating in an end of life care simulation	Voice over PowerPoint lecture prepared by an ENLEC Instructor, end of life care simulation experience, debriefing and guided reflection	Pretest-posttest	Students demonstrated significant increase in knowledge (from pretest mean of 7.98 to posttest mean of 9.15), self-confidence (mean of 6.88, SD 0.61) and self-reported communication skills (mean of 4.33, SD 0.56) in end of life care.
**Pullis** [33] **USA**	To prepare students to care for dying patients and their families.	End of life care orientation lecture and hospice clinical experience as part of a community health nursing course	Not clear	Students were able to demonstrate the: principles of pain and symptom management, ability to communicate the goals and philosophy of hospice care and to advocate for individuals at the end of life

### Results

Our search strategy yielded 778 journal articles of potential use. The search terms used and database results are cited on Table 2. We further identified 2 articles from a manual search of relevant articles. A total of 17 articles met the inclusion criteria. The 17 studies had been conducted in resource-rich countries; USA (13), Canada (1), Australia (1), and UK (1) and assessed the impacts of palliative and end of life care education on student nurses’:

**Table 2:** **Search terms used and results**

Search terms	AND	Database	Number of articles identified excluding duplicates
Palliative care, end of life care, death and dying, terminal care, care of the dying	Nursing Education	CINAHL	430
		EMBASE	309
		MEDLINE	30
		PsychINFO	9
Total			778

Attitudes towards care of the dying [19, 20, 24, 27];

Attitudes and self-perceived competencies in end of life care [28];

Self-efficacy in communication skills and attitudes towards care of the dying [26];

Palliative care knowledge [18, 22];

Knowledge of end of life signs and symptoms, and self-efficacy [31];

Self-perceived comfort level in dealing with dying patients [21],

End of life care knowledge, self-confidence in caring for the dying and self-reported communication skills in working with end of life patients [32] and

Self-perceived competencies in palliative care [29].

The remaining 5 studies described the effectiveness of palliative and end of life care education in improving students’ palliative and end of life care competencies using educational strategies such as workshops [23], films [25] simulated clinical experiences [14, 30] and real life clinical practice experiences [33].

### Educational strategies for Pre-registration palliative care nursing education

#### Course design

Palliative care education at the pre-registration level was delivered in two main ways:

1. Palliative care topics were integrated throughout the nursing program curriculum [24, 25, 33], for example, included in teaching about: quality at the end of life and ethical and legal issues at the end of life in a course about nursing issues and trends; symptom management in a medical/surgical nursing course [24]; and end of life care in a nursing process course [27].
2. Palliative care was offered either as a separate elective [13, 19, 21, 22, 26, 29, 31] or a mandatory course [20, 23].

### Course participants

Participants of preregistration palliative care nursing education included both male and female student nurses of varying ages and cultures and with varying religious views and experiences of death and care of the dying. Most studies involved student nurses in an advanced stage of their program [19, 21, 27, 29, 30] with a minority involving students at an earlier stage [20, 25, 26, 31]. There were two reports of mixed implementation [13, 32] involving students at an advanced stage as well as others who were more junior.

### Course content and duration

In terms of time dedicated to education in palliative care, a wider range from 2 to 50 hours was found. For example, Frommelt [19] implemented a course entitled ‘Living with loss’, which included topics related to loss, dying, death, grief and bereavement, for 45 hours spread over a 15 week semester long period. Mallory [20] implemented an End of Life Nursing Education Consortium (ELNEC) based elective course for 6 weeks. Similarly, Dobbins [27] implemented an elective ‘Nursing Care at the End of Life’ course using the ELNEC curriculum content for 14 weeks. This same author implemented a Nursing Process course that had ELNEC palliative care contents including; pain and symptom management, cultural consideration, patient/family communication, preparation for care at the time of death and after, for 3 hours. Kwekkeboom et al. [22] used a 2 hour session to review palliative care related topics and 4 months for a palliative care companion component. At the other end of the spectrum, Arber [18] implemented an elective end of life care course which included 50 hours of theory and 1 week of hospice placement, delivered over five months.

### Theoretical considerations

Our review shows that preregistration palliative care nursing education is underpinned by transformative and experiential learning theories. With regard to transformative theory, the use of educational strategies like role play, group activities and interaction with patients are reported to encourage critical self-reflection among students to help them evaluate their views and beliefs and to change their attitudes towards care of the dying [20, 24]. Where experiential learning theory is applied, learners’ prior personal experiences, classroom and field based experiences, as well as educational methods that allow learners to interact with and to reflect on the subject matter, are reported by nurse educators to be beneficial to students’ learning about palliative care [13, 22, 23, 25, 28, 30–33].

### Educational approaches

A mix of both didactic and experiential educational strategies is reported. For example, Mallory [20] used a theoretical package alongside experiential learning which took place at a hospice, a funeral home and an anatomy laboratory in order to facilitate transformative learning in students. An approach combining interactive classes with experts, reading of recommended texts, field trips and online discussions was employed by Thompson [21] to enhance students’ learning. In addition to lectures, supplemental texts, hospice and funeral home visits, Dobbins [27] employed a cine- education approach by using a film called ‘Wit’, to educate student nurses about death and dying. Similarly, DiBartolo and Seldomridge [25] asked students who had initially participated in end of life care lectures and had hospice practice experience to watch and reflect on one of two films: *Tuesdays with Morrie* and *Whose Life Is It Anyway?,* to enhance their knowledge of the dying process and the complexity of human reactions when facing end of life situations. Brien et al. [23] used a workshop approach that incorporated emotionally charged learning activities through lectures, clinical case studies, individual and plenary reflective activities, viewing of a documentary film and role-play simulations to enhance the learning of compassionate interventions for end of life care.

Experiential learning strategies such as: using student volunteers as companions of dying patients and their families [22], students’ writing diaries about their palliative care experiences and sharing stories about personal loss [26] clinical simulations using high fidelity patient scenarios [13, 30–32] and real clinical practice experience in an end of life care setting [18, 28, 33], have also been used to educate students about palliative and end of life care.

Furthermore, some nurse educators provided students with supplemental texts, videos and online palliative care educational materials to enrich end of life curricula and bridge the limited presence of end of life care contents in core medical-surgical nursing textbooks [19, 24, 25, 27, 33].

### Outcomes and effectiveness of preregistration palliative care nursing education

#### Research design and sampling

Our review shows that outcomes and effectiveness have been primarily evaluated using quasi-experimental designs, including: a controlled longitudinal design [24], a controlled pretest-posttest design [19, 22, 26, 28], a pretest-posttest single group design [21, 24, 31, 32] and a posttest only design [29, 34]. Some studies employed both qualitative and quantitative methods to assess course impacts and students’ satisfaction [27, 28, 32]. One study used a qualitative design [30]. Other studies did not clearly state how course outcome and/or effectiveness were evaluated [13, 25, 33].

### Outcome measures

Outcome measures included students’ attitude towards death and care of the dying [19, 20, 24, 26, 27], students’ palliative care knowledge [18, 22, 31] and self-perceived competencies in palliative and end of life care [26, 28–31].

### Measurement instruments and methods of data analysis

The assessment of study outcomes were mostly conducted using validated instruments like: the FATCOD (Frommelt Attitude Towards Care of the Dying) scale to assess attitudes towards care of the dying [19, 20, 23, 24, 27], DAP-R scale to measure attitudes towards death [20] and the Palliative Care Quiz for Nursing (PCQN) to assess students’ palliative care knowledge [18, 22]. Nevertheless, instructor-constructed rating scales, without any validity and reliability testing were also used [21, 29, 31]. In the study by Fluharty et al. [32] content validity of developed instruments was established by a group of 12 nurse educators. Additionally, end of course evaluation survey instruments, group interview guides, students’ reflective journals and/or examination scores have been used by some authors to assess students’ satisfaction with the educational activity, self-perceived course outcomes and effectives, as well as students’ perspectives on course improvement strategies, where applicable [23, 25, 31].

Inferential statistical analysis methods were predominantly utilised in the data analysis [19, 20, 22, 27, 29, 31, 32]. Studies with smaller samples used descriptive statistics [26]. Thematic analysis was utilised by the qualitative studies [13, 30].

### Reported outcomes and effectiveness

Some studies reported a positive impact of palliative care education [19, 20, 24, 26, 27, 31], especially in improving attitudes towards care of the dying. For example Barrere et al. [24] recorded a significant overall change in students’ attitudes towards care of the dying (t = -5.977, p = .000). In addition, Weismann [26] also registered a 6 point increase in attitudes towards care of the dying in an intervention group compared to 0.6 point increase in a control group. Similarly, Thompson [21] found a significant improvement in comfort levels in dealing with issues regarding the care of dying patients and their family, from an average of 4.8 on a scale of 10 to an average of 7.5. However, variables like age, gender, previous death experience, and religious beliefs are reported as predictors of attitude change [19, 20, 24]. In the study by Barrere et al. [24], lack of previous experience with death and an age of 18–22 accounted for the most variance in attitude change.

Of the two studies in this review that investigated the impact on knowledge using the PCQN Questionnaire, which is a validated instrument, one recorded no significant knowledge improvements [22] and the other [18] reported significant improvements in students’ knowledge of symptom control and opioid use. On the other hand, while Fluharty et al. [32] and Moreland et al. [31] both reported significant improvements in students’ end of life care knowledge, the increase in the study by Moreland et al. [31] was particularly related to knowledge of end of life symptoms. Pullis [33] states that students were able to demonstrate: the principles of pain and symptom management, the ability to communicate the goals and philosophy of hospice care and the ability to advocate for individuals at the end of life, but does not provide any evidence of measures used in this assessment.

Weismann [26] reported positive impacts on self-efficacy in communication skills; from a pretest mean of 61.7 (SD = 25.86), to a posttest mean of 80.0 (SD = 15.2). Similarly, Moreland et al. [31] and Fluharty et al. [32] also recorded a significant increase (p = .05 and p = .000 respectively) in students’ self-efficacy in end of life care. Notwithstanding, Gilliland [28] did not record any significant change in self-reported competencies in end of life care. On a general scale however, these studies report that palliative and end of life care education is effective in improving student nurses’ self-perceived competencies in palliative and end of life care [13, 23, 26, 29, 30, 33].

### Discussion

There seems to be some tension regarding whether or not palliative care content should be embedded throughout the entire pre-registration curriculum or taught as a discrete course. While Pullis [33] states that the embedded content approach can facilitate the incorporation of palliative care into the curriculum without adding to what may already be extensive material, some authors argue that a discrete course is more beneficial because it allows for better assimilation of material by students [18, 20]. While there is evidence of the effectiveness of both approaches in improving student nurses’ attitudes towards death and care of the dying [27], embedding palliative care content into other nursing courses or offering a discrete course as an elective might not give palliative care nursing education the attention it deserves. In addition, the lack of evidence of the effectiveness of a discrete course over the embedded content approach warrants more research.

Whether students should be exposed to palliative and end of life care content at an earlier or later stage of their nursing course remains unclear. While it is suggested that at a later stage students might have some background knowledge which facilitates their learning about palliative care [20], earlier implementation might enhance students’ understanding of the principles of palliative care and prevent them from developing misconceptions about palliative care during early clinical practice experiences [20, 34]. This seems to suggest that decisions about when to include palliative care education at the pre-registration level should be informed by students’ previous learning and clinical practice exposure.

This review reveals a lack of consistency in what pre-registration student nurses are taught in palliative care, even when these courses have been implemented in nursing schools in the same country and/or using the same core curriculum. In addition, there continues to be inadequate evidence on the number of hours and period of time over which content should be distributed as had been observed by Arber [18]. There tends to be either more focus on clinical experience without adequate theoretical content or vice versa. Nurse educators ideally need to ensure that palliative care content taught at the pre-registration level is in line with international and regional palliative care core curricula [35–37]. Nevertheless, it is acknowledged that contextual realities as well as epidemiological and demographic trends fuelling the need for palliative care can require certain content and teaching method amendments.

A mix of both didactic and experiential teaching and learning strategies, as well as the use of specialist and experienced palliative care lecturers, have been shown to be invaluable in enhancing students learning of palliative care contents. Furthermore, the relevance of providing students with opportunities to care for dying patients in supervised simulated and real life situations is highlighted, thus suggesting that delivery of theoretical content without exposure to practice is inadequate.

Based on the studies reviewed, we might want to conclude that pre-registration palliative care education is effective in improving student nurses’ attitudes towards death and the care of dying patients and their families. However, the predominant use of non-probability sampling methods and employment of self-rating evaluation instruments in these studies might have introduced some biases. In addition, variables like age, gender, previous death experience and religious beliefs have been reported as predictors of attitude change [19, 20]. Moreover, results of those studies without a control group may have been affected by extraneous variables [38]. In addition, the use of small sample sizes while beneficial to the educational process [34] raises methodological issues relating to representativeness and generalizability of results.

While this review has provided some evidence about the effectiveness of palliative care education on preregistration student nurses’ competencies in palliative care, there seems to be little or no evidence about transfer of learning to clinical practice by these students, and what the facilitators and barriers to this transfer might be. In addition, there are no reports of the impacts of education on patients’ care experiences. The lack of such evidence tends to blind us to any potential short or long term benefits that preregistration palliative care education might have on patients and their families.

Furthermore, the dearth of in-depth qualitative studies means that little is known about the palliative care education experiences of preregistration students; these could inform the improvement of current educational strategies. Overall, there has been inadequate evaluation of the outcome of palliative care educational initiatives warranting further research.

Given that all the studies in this review were conducted in resource-rich countries, we shall now examine the implications of these findings in resource-poor countries.

### Assessment of Implications for Curriculum Development in Resource Poor Countries

#### A) Course design and content

A discrete compulsory palliative care course for pre-registration student nurses seems more suitable for resource-poor contexts, compared to embedding palliative care contents into other nursing speciality courses or providing it as an elective. This is because there are only a few specialist palliative care professionals and institutions in resource-poor contexts [9, 12]. In addition, the huge burden of HIV/AIDS [39], cancer and other non-communicable chronic diseases in this context [40], coupled with the fact that most of palliative care in this context is often delivered by general nurses in hospitals and in the domestic home [2] requires every nurse to be educated in palliative care. This can ensure that every dying person and their families have access to basic palliative care, which has been termed a basic human right [41]. The adoption of a discrete compulsory course however needs to be complemented with strict psycho-emotional support to ease any emotional burdens that might be experienced by participating students. With regards to determining the content to be taught, a review of regional and/or international palliative care core curricula documents, and an examination of local demographic and epidemiologic challenges, as well as social and cultural realities would inform the development of an appropriate content. As observed by Spruyt, MacLeod and Hudson (2007:67) [42]: “creative and culturally specific responses to the palliative care education challenge are more likely to succeed”.

#### B) Educational methods

Most of the proposed educational strategies would seem applicable in resource-poor countries. However, there are challenges in the availability of specialist palliative care units for placement learning, affordable palliative care nursing textbooks and journals [43, 44] and few funds to develop and implement high fidelity patient simulations. Nevertheless, in the absence of hospices and/or specialist palliative care hospital units, students can be exposed to meaningful learning opportunities in medical-surgical, paediatric and intensive care units in local hospitals and health centres, that provide care to patients with life-threatening illnesses [2, 45, 46].

#### C) Expected outcomes and evaluation

In designing course objectives and evaluation strategies, there is need to address the cognitive, affective and psychomotor domains of learning. This will enhance understanding of how palliative care education impacts on student nurses’ knowledge, altitudes and skills [46]. It is also crucial that strategies evaluating both the process and outcomes of education are considered [18]. Drawing from Kirkpatrick’s [47] framework of training program evaluation and in keeping with ongoing theory to practice transfer debates [48] a comprehensive evaluation of a pre-registration palliative care nursing program should ascertain both students’ satisfaction with, and learning from the program, and their ability to translate this knowledge into behaviour that can make a positive difference to patients and their families.

#### D) Course faculty

Another major challenge in resource-poor countries is the lack of availability of specialist and experienced palliative care lecturers. Where nurses who have had specialist palliative care education or have attended ‘train the trainer’ courses and palliative care conferences are available, they could possibly serve as course faculty. However, where there are no formally trained professionals it could be argued, based on the experiential Kolb [49] and adult learning theories Knowles et al. [50] that nurse educators and clinicians with wider experience in the care of patients with life-threatening conditions or the dying should serve as facilitators as long as they have access to core palliative care textbooks and other types of resources. This strategy however requires careful consideration to ensure that students are engaged with appropriate palliative care information and evidence based practice. In addition, with the availability of funding, expert faculty could be invited from overseas institutions. Still, the challenge as observed by Goh and Shaw [51] might be the faculty’s lack of understanding of the cultural and contextual realities. These might lead to students being oriented to practices which are unavailable or inappropriate in their cultural context.

## Conclusion

This modified systematic review about pre-registration palliative care education has revealed that palliative care education can be effective in improving student nurses’ attitudes towards care of dying patients and their family. At this level, palliative care education is delivered either as a discrete course or is embedded in other specialty nursing courses. Both didactic and experiential educational strategies are employed and education is variably delivered to students at earlier or advanced stages of their nursing program.

These educational strategies, which have been tried mostly in resource-rich countries, need to be adapted for resource-poor countries to bridge barriers such as the lack of specialist palliative care practitioners who can serve as educators, absence of specialist palliative care units in hospitals and community health settings for experiential learning, and problems in access to palliative care textbooks, journal and online educational materials.

Based on the findings of this review, a 30 hour classroom based course in palliative care has been developed and piloted by the lead author with pre-registration student nurses of the University of Buea in Cameroon. This course, which is underpinned by experiential learning theory, was delivered by nurse educators and palliative care nurses in Cameroon to second and third year student nurses. The evaluation of the impact of the course on students’ palliative care knowledge and self-perceived competence and confidence in palliative care provision is underway.

### What is known about this topic?

Nurses lack competence and confidence in palliative care. There is need for pre-registration student nurses to be educated in palliative care, prior to entering the profession. Although palliative care content is beginning to feature in pre-registration nurse training curricula in some resource-rich countries, there is still a significant lack of presence in the curricula of resource-poor countries.

### What this paper Adds?

Pre-registration palliative care education has been delivered either as a discrete course or as embedded contents in other specialty nursing courses, using both didactic and experiential educational strategies, to students who are either at an earlier or advanced stage of their nursing program. Education can be effective in improving student nurses’ attitudes towards care of dying patients and their family. Suggestions are made for the development of preregistration palliative care education in resource-poor countries.

## Authors’ information

Nahyeni Bassah is a Nurse Educator and teaches undergraduate student nurses at the University of Buea and some Higher Institutes of learning in Cameroon. She is currently a PhD student with the School of Health Sciences, University of Nottingham, United Kingdom.

Jane Seymour is Sue Ryder Care Professor in Palliative and End of Life Studies at the School of Health Sciences, University of Nottingham, United Kingdom and is Nahyeni’s PhD supervisor.

Karen Cox is a Professor of Cancer and Palliative Care in the School of Health Sciences, University of Nottingham, United Kingdom and is Nahyeni’s PhD supervisor.
